# Pharmacological Perturbation of the Golgi Checkpoint Synergizes with Apoptotic Priming to Selectively Induce Tumor Cell Death

**DOI:** 10.3390/cells15191825

**Published:** 2026-10-07

**Authors:** Roberta Iannitti, Antonino Colanzi, Fabiola Mascanzoni

**Affiliations:** Institute of Endotypes in Oncology, Metabolism, and Immunology “G. Salvatore” (IEOMI), Street Pietro Castellino 111, 80131 Naples, Italy

**Keywords:** Golgi checkpoint, apoptotic priming, combinatorial drug strategy

## Abstract

Proper mitotic progression requires not only completion of DNA replication but also structural remodelling of the Golgi complex, including unlinking of the Golgi ribbon into isolated stacks during G2. This process activates a Golgi-dependent checkpoint that ensures accurate spindle assembly and cytokinesis. We previously showed that preventing GRASP65 phosphorylation through JNK inhibition blocks Golgi unlinking, impairs Aurora-A recruitment to centrosomes and, upon forced mitotic entry, induces multipolar spindles and binucleation. These studies established the mechanistic framework linking Golgi unlinking to mitotic entry and spindle formation. Here, building on this established framework, we asked whether the resulting mitotic vulnerability could be pharmacologically exploited. We therefore used SP600125 (SP) together with MK1775 (MK) as a previously characterized pharmacological platform and screened for compounds capable of further sensitizing these cells to death. This screen identified Navitoclax, a BCL-2/BCL-XL inhibitor, as the strongest enhancer of the SP+MK response. The triple combination selectively inhibited proliferation and induced apoptosis in tumor cells, while non-transformed RPE-1 cells remained largely resistant. Importantly, its activity depended on Golgi integrity: disrupting Golgi architecture with Brefeldin A partially restored cell viability, and efficacy correlated with Golgi compaction. Annexin V analysis confirmed apoptosis rather than mitotic slippage. Together, these findings identify a tumor-selective vulnerability linked to Golgi checkpoint disruption and support a therapeutic strategy combining Golgi checkpoint inhibition, forced mitotic entry and apoptotic priming to selectively eliminate cancer cells.

## 1. Introduction

Entry into mitosis is governed not only by the completion of DNA replication but also by the fulfillment of structural and signalling prerequisites that occur during G2. Among these, Golgi unlinking plays a crucial role by separating the interconnected Golgi stacks into individual units. This structural remodeling is not merely a passive reorganization: it acts as a functional checkpoint that ensures proper centrosome maturation, spindle assembly, and mitotic fidelity [1]. Block of unlinking results in the activation of a Golgi-dependent checkpoint that halts cell cycle progression in G2 [2].

A key molecular mediator of this checkpoint is the Golgi Reassembly and Stacking Protein 65 (GRASP65), a structural matrix protein that tethers Golgi cisternae and stabilizes Golgi-associated microtubules (MT). During G2, GRASP65 is phosphorylated at serine 274, a modification primarily mediated by the JNK2 kinase, which leads to loss of its tethering and MT-stabilizing functions, thereby promoting Golgi unlinking [3]. This phosphorylation is part of a broader signalling cascade that includes PLK1 and MEK/ERK pathways, all of which converge to remodel Golgi architecture in preparation for mitosis [4]. We have previously shown that the failure of Golgi unlinking in G2, achieved by inhibiting GRASP65 phosphorylation via JNK2 blockade, causes a potent G2 arrest. Forcing such arrested cells into mitosis using the Wee1 inhibitor MK1775, which activates CDK1, results in severe mitotic defects, including multipolar spindles and binucleation [5].

Mechanistically, these phenotypes depend on the insufficient recruitment of Aurora-A kinase to the centrosomes, as unlinking normally triggers Src-mediated phosphorylation of Aurora-A at tyrosine 148, a modification essential for its centrosomal localization and activation [6].

These findings define a Golgi checkpoint that integrates architectural status with Src-Aurora A activation to coordinate mitotic entry. Notably, the Golgi is the only organelle whose structural remodeling is actively monitored before mitosis [7], underscoring the specificity and potential exploitability of this checkpoint.

Given that tumor cells frequently display spindle abnormalities, chromosomal instability, and increased reliance on mitotic surveillance pathways, we hypothesized that they may be selectively vulnerable to perturbations of the Golgi checkpoint. Our previous studies established a mechanistic framework in which JNK2-dependent GRASP65 phosphorylation promotes Golgi unlinking during G2, which in turn supports Golgi-dependent Aurora-A recruitment and proper spindle formation [3,5,6]. The present study was designed to determine whether the mitotic vulnerability created by perturbing this established pathway could be pharmacologically exploited. Accordingly, we used the previously characterized SP600125+MK1775 treatment as a pharmacological platform: SP600125 inhibits JNK-dependent Golgi unlinking, whereas MK1775 overrides the resulting G2 arrest and forces cells into defective mitosis [5]. Building on this SP+MK backbone, we systematically screened a panel of small molecules targeting mitotic regulators (Aurora kinases, PLKs, and spindle assembly factors) and apoptotic modulators to identify compounds that enhance mitotic stress and selectively compromise viability in cancer cells. Among the candidates tested, Navitoclax, a BCL-2/BCL-XL inhibitor [8], emerged as the most potent sensitizer of SP+MK-induced cell death. Strikingly, this effect was preferentially observed in tumor cells, while non-transformed cells remained largely refractory under the same treatment conditions, suggesting that Navitoclax amplifies a Golgi checkpoint-dependent vulnerability by lowering the apoptotic threshold in cells already burdened by mitotic dysfunction.

Together, our findings indicate that combining Golgi unlinking inhibition, forced mitotic entry, and apoptotic priming constitutes a tumor-selective strategy that converts the Golgi checkpoint-dependent mitotic stress into apoptotic cell death in cancer cells. These results provide a proof-of-concept for Golgi checkpoint-targeted combinatorial therapies.

## 2. Materials and Methods

### 2.1. Cell Culture and Treatments

HeLa cells (ECACC 93021013) were cultured in Dulbecco’s Modified Eagle Medium (DMEM, Gibco, Waltham, MA, USA) supplemented with 10% fetal bovine serum (FBS, Gibco, Waltham, MA, USA), 2 mM L-glutamine (Lonza, Verviers, Belgium), and 50–100 units/mL penicillin/50–100 µg/mL streptomycin. hTERT-RPE1 cells (kindly provided by B. Franco, Telethon Institute of Genetics and Medicine, TIGEM, Naples, Italy) were grown in DMEM/F12 supplemented with 10% fetal bovine serum (Gibco), 2 mM L-glutamine, 1 U/mL penicillin, and 50 µg/mL streptomycin (Invitrogen, Frederick, MD, USA). All cell lines were maintained at 37 °C in a humidified atmosphere containing 5% CO_2_. For all experiments, cells were seeded at approximately 60% confluence and treated 24 h later with the indicated compounds, either alone or in combination.

### 2.2. Antibodies and Reagents

Thymidine (2 mM), SP600125 (25 μM), JNK inhibitor III (5 μM), Brefeldin A (200 ng/mL), fibronectin (10 μg/mL), and Crystal Violet were purchased from Sigma-Aldrich (St. Louis, MO, USA). MK1775 (0.3 μM), Navitoclax (0.5 μM) and all drugs cited in Table 1 were from Selleckchem. DMSO was from Carlo Erba. Hoechst 33342 was from Invitrogen. Mowiol 4-88 was from Sigma-Aldrich. Incucyte Annexin V NIR Dye was from Sartorius (cat. 4768).

### 2.3. Count of Nuclei

HeLa cells were seeded in 24-well plates, synchronized in S phase by double thymidine block (2 mM thymidine, two consecutive blocks) as previously described in [5], and treated with the indicated compounds for 24 h. Cells were then fixed with 4% paraformaldehyde and stained with Hoechst 33342. Nuclei were counted in ten randomly selected fields per condition using a fluorescence microscope. Data are representative of three independent experiments.

### 2.4. Crystal Violet Assay

For cell viability assessment, 4 × 10^4^ HeLa cells were seeded per well in 24-well flat-bottom plates in 1 mL of medium, allowed to adhere overnight, and synchronized by double thymidine block as described above. Cells were then treated overnight with the indicated compounds, alone or in combination. After treatment, medium was removed and cells were washed with PBS and fixed and stained with 0.1% (*w*/*v*) crystal violet in 25% methanol for 30 min in the dark. Stain was removed, cells were washed twice with double-distilled water and allowed to dry. Crystal violet was solubilized with 10% (*v*/*v*) acetic acid and absorbance was measured at 595 nm using a Cytation 3 imaging reader (Agilent BioTek, Winooski, VT, USA). Data are expressed as mean ± SD of three independent experiments.

### 2.5. IncuCyte Live-Cell Imaging

Cell proliferation was continuously monitored using the IncuCyte S3 Live-Cell Analysis System (Sartorius, Ann Arbor, MI, USA). Cells were seeded in 24-well plates (20,000 cells per well) and treated with the indicated compounds. Four sets of phase-contrast images from distinct regions within each well were acquired every 4 h over a 72 h period using a 10× objective. Cell confluence was determined using the integrated IncuCyte S3 image analysis software and normalized to the initial value at time zero to account for plating variations. Data are representative of three independent experiments, each performed in triplicate.

### 2.6. Real-Time Apoptosis Assay

Apoptotic activity was monitored using the IncuCyte S3 Live-Cell Analysis System (Sartorius). HeLa cells were seeded in 24-well plates (20,000 cells per well), treated with the indicated compounds, and incubated with Annexin V NIR Dye (Sartorius, cat. 4768) for the duration of the experiment. Four sets of phase-contrast and NIR fluorescence images were acquired every 4 h over 72 h using a 10× objective. Apoptotic activity was quantified as the ratio of Annexin V NIR-positive area to phase-contrast area, normalized to the value at time zero for each well. Data are representative of three independent experiments, each performed in triplicate.

## 3. Results

### 3.1. A Compound Screening Identifies Navitoclax as a Potent Sensitizer of Golgi Checkpoint-Induced Mitotic Stress

The aim of this study was to evaluate whether the mitotic vulnerability induced by Golgi checkpoint perturbation could be pharmacologically amplified; we screened a panel of small molecules in combination with SP600125 (SP) and MK1775 (MK). Compounds were selected to target pathways mechanistically linked to Golgi-dependent mitotic control, including Aurora kinases, PLKs, spindle assembly factors, and apoptotic regulators. All agents were tested both as single treatments and in combination with SP+MK, and their effects on cell proliferation were quantified by nuclei count immunofluorescence in HeLa cells. Drug concentrations were selected based on previously published in vitro studies in human cell lines, within the range of pharmacologically active doses reported in the literature (see Table 1 for details). The compounds tested, along with their respective targets, pathways, and concentrations, are summarized in Table 1. HeLa cells were synchronized by double-thymidine block [5] and treated overnight with SP+MK (25 µM and 0.3 µM, respectively) alone or in combination with each compound at concentrations below their single-agent antiproliferative threshold. Cell proliferation was assessed by Hoechst staining and nuclei counting per field of view (f.o.v) by fluorescence microscopy.

We first tested inhibitors of Aurora kinases, given their established roles in centrosome maturation, spindle formation, and cytokinesis: processes directly impaired by Golgi checkpoint perturbation [9,10,11]. Alisertib (Aurora A inhibitor) and AT9283 (Aurora A/B inhibitor) both produced a modest but statistically significant additive reduction in cell proliferation when combined with SP+MK (Figure 1A). In contrast, Danusertib, a pan-Aurora kinase inhibitor (Aurora A/B/C) [12], did not enhance the antiproliferative effect of the SP+MK combination compared to SP+MK alone. We next evaluated inhibitors targeting the spindle assembly machinery, including Ispinesib (KSP inhibitor), AZ3146 (Mps1/SAC inhibitor), and Monastrol (Eg5 inhibitor) [13,14,15]. These agents were selected based on their ability to interfere with spindle dynamics and chromosome segregation, processes likely to be further impaired upon premature mitotic entry. However, none of the three compounds enhanced the antiproliferative effect of SP+MK when tested in combination, and cell proliferation remained comparable to SP+MK alone for all three agents (Figure 1B). We then tested microtubule-targeting agents, including Vinblastine (microtubule depolymerizer) [16] and Paclitaxel (microtubule stabilizer) [17] together with Rigosertib [18], a compound that has been shown to induce mitotic spindle abnormalities, although its primary mechanism of action remains debated [19]. None of these agents produced a significant enhancement of the SP+MK antiproliferative effect when tested in combination, with cell proliferation remaining comparable to SP+MK alone in all cases (Figure 1C). We also tested a panel of DNA-damaging and antimetabolite agents, such as Methotrexate (DHFR inhibitor), 5-Fluorouracil (thymidylate synthase inhibitor), Cisplatin (DNA crosslinker), and Doxorubicin (topoisomerase II inhibitor and DNA intercalator), reasoning that increased replication stress might sensitize cells to subsequent mitotic perturbation [20,21,22,23]. However, none of these agents enhanced the antiproliferative effect of SP+MK when tested in combination (Figure 2A). Given the established role of MEK/ERK signaling in Golgi fragmentation during mitotic entry, we next tested a panel of MEK inhibitors, including Binimetinib, Selumetinib, Trametinib, and TAK733, as well as Sorafenib, a multikinase inhibitor targeting RAF and downstream MAPK signaling [24,25,26,27,28]. However, none of these agents enhanced the antiproliferative effect of SP+MK when tested in combination, with cell proliferation remaining comparable to SP+MK alone in all cases (Figure 2B). Since none of the mitotic regulators tested enhanced SP+MK-induced antiproliferative effects, we shifted our focus to modulators of apoptotic signalling, beginning with GSK2256098, a FAK inhibitor selected for its ability to suppress JNK and ERK signalling, pathways directly implicated in Golgi checkpoint regulation [29]. However, GSK2256098 did not enhance the antiproliferative effect of SP+MK when tested in combination (Figure 2C). In contrast to the mitotic regulators tested above, Navitoclax, a BCL-2/BCL-XL inhibitor selected for its ability to lower the apoptotic threshold in mitotically stressed cells, produced a striking enhancement of SP+MK-induced antiproliferative effects [5,30]. The triple combination of Navitoclax, SP, and MK resulted in a marked reduction in cell proliferation compared to SP+MK alone and to each single agent, as quantified by nuclei counting (Figure 2D). Collectively, these results identified Navitoclax as the only compound capable of significantly enhancing SP+MK-induced antiproliferative activity, and established the triple combination as the most effective regimen treatment strategy emerging from this screening.

**Table 1:** Compounds tested in combination with SP600125 and MK1775. For each compound, the respective molecular target(s), pathway/function, and concentration used are indicated.

Compound	Target(s)	Pathway/Function	Concentration Tested	References
AT9283	Aurora A/B	Centrosome maturation/spindle assembly/chromosome condensation	5 µM	PMID:20507304 [11]
Alisertib	Aurora A	Centrosome maturation/spindle assembly	20 µM	PMID:22016509 [9]
Danusertib	Aurora A/B/C	Centrosome maturation/spindle assembly/chromosome condensation	1 µM	PMID:18089710 [12]
Ispinesib	KSP	Spindle assembly	2 µM	PMID:20068098 [13]
AZ3146	Mps	Chromosome alignment/Spindle assembly	1.2 µM	PMID:20624899 [14]
Monastrol	Kinesin-5	Spindle assembly	6 µM	PMID:10973989 [15]
Rigosertib	PLK1	Centrosome maturation/spindle assembly	1 µM	PMID:15766665 [18]
Vinblastin	Tubulin depolymerization	Mitotic arrest/microtubule disruption	10 µM	PMID:35955645 [16]
Paclitaxel	Tubulin stabilization	Spindle assembly/Mitotic checkpoint	0.5 µM	PMID:19720871 [17]
Methotrexate	Dihydrofolate reductase (DHFR)	DNA synthesis	0.5 µM	PMID:34295794 [20]
5-fluouraracil	Thymidylate synthase (TS)	DNA replication	2.5 µM	PMID:31623083 [21]
Doxorubicin	Topoisomerase II	DNA intercalation/DNA damage	1 µM	PMID:22593250 [23]
Cisplatin	Binding of Adenine and Guanine	DNA intercalation/DNA damage	5 µM	PMID:33333716 [22]
GSK2256098	Focal Adhesion Kinase (FAK)	Anchorage-independent growth	5 µM	PMID: 25486573 [29]
Navitoclax	BCL-2, BCL-xL	Apoptotic priming	0.5 µM	PMID: 18451170 [30]
TAK733	MEK1/2	Proliferation	1 µM	PMID:30396931 [27]
Binimetinib	MEK1/2	Proliferation	1 µM	PMID:28152546 [24]
Selumetinib	MEK1/2	Proliferation	10 µM	PMID:20587667 [25]
Trametinib	MEK1/2	Proliferation	0.5 µM	PMID:22663011 [26]
Sorafenib	RAF/MAPK pathway	Proliferation	10 µM	PMID:15466206 [28]

### 3.2. Brefeldin A-Induced Golgi Dispersal Attenuates the Antiproliferative Effect of the Navitoclax+SP+MK Combination

Next, we sought to determine whether the antiproliferative effect of the Navitoclax+SP+MK combination depends on Golgi integrity. We disrupted Golgi architecture using Brefeldin A (BFA), which abolishes the structural prerequisite for SP+MK-induced mitotic defects [5,31]. Importantly, the effects of SP+MK treatment, both alone and in combination with BFA, on Golgi morphology have been previously documented by immunofluorescence microscopy (Figure 1B and Figure 2D; Mascanzoni et al. [5]).

HeLa cells were treated with the triple combination in the presence or absence of BFA, and cell proliferation was assessed by nuclei counting and crystal violet staining (Figure 3A–C). BFA treatment resulted in a significant, albeit partial, rescue of cell viability compared to the triple combination alone. The incomplete nature of this rescue is consistent with the known cytotoxicity of prolonged BFA exposure [32] rather than reflecting residual Golgi checkpoint activity. These results support a contribution of Golgi integrity to the antiproliferative efficacy of the Navitoclax+SP+MK combination and are consistent with the involvement of the previously characterized Golgi checkpoint pathway.

### 3.3. Differential Sensitivity of Tumor and Non-Transformed Cells to SP+MK Treatments

Tumor cells are generally more susceptible than non-transformed cells to mitotic arrest induced by drugs targeting mitotic regulatory pathways and promoting cellular stress accumulation [33]. To assess whether tumor cells are selectively sensitive to Golgi checkpoint perturbation, we compared the response of HeLa and RPE-1 cells to SP+MK treatment by monitoring cell survival over 72 h using the IncuCyte S3 live-cell imaging system (Figure 4). Survival was normalized to the untreated control for each cell line. HeLa cells were highly sensitive to SP+MK treatment, with more than 80% cell death observed at 72 h, whereas RPE-1 cells were largely refractory, maintaining nearly 100% survival throughout the experiment. These results demonstrate that blocking Golgi unlinking and forcing premature mitotic entry selectively compromises viability in tumor cells, while non-transformed cells tolerate the same treatment, consistent with their more robust checkpoint control and lower intrinsic mitotic stress.

### 3.4. Navitoclax Selectively Potentiates SP+MK-Induced Cell Death in Tumor Cells

To further investigate the contribution of Navitoclax to the SP+MK treatment, we monitored cell proliferation in HeLa and RPE-1 cells over a 72 h time course using the IncuCyte Zoom live-cell imaging system. The triple combination of Navitoclax (0.5 µM), SP600125 (25 µM), and MK1775 (0.3 µM) significantly reduced proliferation in HeLa cells compared with each drug administered alone (Figure 5A, see red line), whereas the same treatment had only a minimal effect on non-transformed RPE-1 cells (Figure 5B). To improve readability, SD error bars were omitted from selected panels in Figure 5. For completeness, Supplementary Figure S1 presents the same graphs with all SD error bars included. To further quantify this differential response, cell survival was normalized to untreated controls and plotted as cumulative survival curves. HeLa cells were highly sensitive to the triple combination, exhibiting a reduction in survival of more than 80% at 72 h, whereas RPE-1 cells maintained nearly complete viability throughout the experiment.

These findings indicate that Navitoclax enhances the anti-proliferative activity of the SP+MK combination selectively in cancer cells (Supplementary Figure S2). The preferential sensitivity of HeLa cells may reflect vulnerabilities commonly associated with malignant transformation. Cancer cells frequently experience elevated levels of oncogenic, replicative, and genotoxic stress, which increase their dependence on anti-apoptotic BCL-2 family proteins for survival, a phenomenon referred to as apoptotic priming [34]. In parallel, defects in cell-cycle checkpoint pathways render tumor cells particularly susceptible to WEE1 inhibition, which promotes premature mitotic entry in the presence of unresolved DNA damage, thereby exacerbating replication stress and mitotic catastrophe [35]. Although the molecular basis of the selective response observed here remains to be fully elucidated, our results are consistent with a model in which Navitoclax cooperates with SP600125 and MK1775 to exploit both the heightened apoptotic priming and the increased sensitivity to cell-cycle perturbation that characterize cancer cells. The limited effect observed in RPE-1 cells suggests the existence of a potential therapeutic window for this combinatorial strategy [36].

### 3.5. The Antiproliferative Efficacy of the Navitoclax+SP+MK Combination Is Preserved at Reduced Drug Concentrations

We next asked whether the antiproliferative efficacy of the triple combination was maintained at lower drug concentrations. HeLa cells were treated with Navitoclax (0.5 µM) combined with reduced doses of SP600125 (10 µM) and MK1775 (0.1 µM), and cell proliferation was assessed by nuclei counting and crystal violet staining (Figure 6A,B). Both assays demonstrated a significant reduction in cell proliferation, comparable to that observed at standard doses. This was further confirmed by IncuCyte S3 live-cell imaging over a 72 h time course, which showed that the triple combination at reduced doses significantly decreased proliferation in HeLa cells while producing no comparable effect in RPE-1 cells (Figure 7A,B); (Supplementary Figure S3 for details). These results indicate that the tumor-selective antiproliferative activity of the combination is maintained even at lower concentrations of SP and MK, suggesting a potential therapeutic window that may be exploitable at clinically tolerable doses.

### 3.6. The Antiproliferative Efficacy of the Triple Combination Correlates with the Degree of Golgi Compaction

To further assess the contribution of Golgi unlinking inhibition to the triple combination effect, we tested JNK inhibitor III (5 µM), a compound that compacts the Golgi ribbon less effectively than SP600125 [3], in combination with MK1775 and Navitoclax in HeLa cells, monitoring cell proliferation by IncuCyte S3 live-cell imaging over a 72 h time course (Figure 8) (Supplementary Figure S4 for details). The Navitoclax+JNK III+MK1775 combination reduced cell proliferation compared to single agents, but to a lesser extent than Navitoclax+SP600125+MK1775, consistent with the comparatively weaker Golgi compaction induced by JNK III. Together with the BFA rescue experiment, these findings are consistent with the antiproliferative response being influenced by the extent of Golgi architectural perturbation, in agreement with the Golgi checkpoint framework established in our previous studies.

### 3.7. Navitoclax Increases Apoptotic Cell Death in SP+MK-Sensitized Cells

To directly assess whether Navitoclax promotes apoptotic cell death in SP+MK-treated cells, we monitored apoptosis induction using an Annexin V assay on the IncuCyte S3 live-cell imaging system over a 72 h period in HeLa cells [37]. The triple combination Navitoclax+SP+MK produced a significant increase in Annexin V-positive cells compared to each single agent and to SP+MK alone (Figure 9); (Supplementary Figure S5). These results demonstrate that the enhanced antiproliferative effect of the triple combination is associated with increased apoptotic activity, consistent with a model in which Navitoclax lowers the apoptotic threshold in cells sensitized by SP+MK-induced, Golgi checkpoint-dependent mitotic stress, thereby increasing apoptotic cell death.

## 4. Discussion

The present study extends our previous mechanistic characterization of the Golgi checkpoint by asking whether the mitotic vulnerability generated by its perturbation can be pharmacologically exploited to enhance tumor cell killing. Our earlier studies established the molecular framework linking JNK2-dependent GRASP65 phosphorylation and Golgi unlinking to Golgi-dependent Aurora-A recruitment and proper spindle formation during mitotic entry [3,5,6]. On this basis, the SP600125+MK1775 combination was used here as a pharmacological platform to generate Golgi checkpoint-dependent mitotic stress, rather than to redefine the underlying molecular pathway.

Within this framework, our main finding is that lowering the apoptotic threshold with Navitoclax strongly enhances the antiproliferative and apoptotic response to SP+MK treatment. The conceptual advance of the present study is the connection between an established Golgi checkpoint-dependent mitotic vulnerability and pharmacological apoptotic priming.

Notably, the differential sensitivity of HeLa and RPE-1 cells to SP+MK treatment is consistent with the established notion that tumour cells are more susceptible to mitotic perturbations than non-transformed counterparts [33]. This vulnerability likely reflects the defective checkpoint control and elevated intrinsic stress that characterize cancer cells, reducing their capacity to tolerate additional mitotic insults. In this context, compromised checkpoint integrity may also enhance susceptibility to Wee1 inhibition by MK1775, which promotes premature mitotic entry and thereby exacerbates cellular stress [35]. Concurrently, cancer cells can exhibit increased dependence on anti-apoptotic BCL-2 family members and altered mitochondrial apoptotic priming, potentially increasing their sensitivity to Navitoclax-mediated inhibition of these survival pathways [34,38]. These mechanisms were not directly investigated in the present study and therefore represent plausible, rather than definitive, explanations for the differential response observed between HeLa and RPE-1 cells. Together, these vulnerabilities may contribute to the preferential sensitivity of HeLa cells to the triple combination and suggest the existence of a potential therapeutic window in the experimental system examined here. Two observations in the present study further support the involvement of Golgi architecture in the antiproliferative response to the triple combination. First, Brefeldin A-mediated disruption of Golgi architecture partially rescued cell viability in Navitoclax+SP+MK-treated cells, consistent with a contribution of an intact Golgi to the response. Second, substitution of SP600125 with JNK inhibitor III, which induces weaker Golgi compaction [3], produced a less pronounced antiproliferative effect. Together, these observations provide internal pharmacological support for the proposed interpretation and are consistent with the Golgi checkpoint framework established by prior genetic, biochemical, and rescue studies [3,5,6].

Finally, Annexin V profiling provided evidence consistent with increased apoptotic activity following treatment with the triple combination. These findings support a model in which Golgi checkpoint perturbation generates mitotic stress that contributes to apoptotic cell death, while Navitoclax lowers the apoptotic threshold required to convert this stress into cell death [37].

This observation supports a model in which Golgi checkpoint perturbation generates mitotic stress that primes cells for apoptotic elimination, while Navitoclax lowers the apoptotic threshold required to convert that stress into cell death. Accordingly, the present study should be viewed as a pharmacological proof-of-concept connecting the mechanistic foundation established in our previous work with future validation in disease-relevant and preclinical models. It remains to be determined whether the observed tumor selectivity extends to cancer models with distinct molecular backgrounds, BCL-2 family dependencies, and checkpoint status. Such studies should include genetic dissection of the contribution of individual anti-apoptotic BCL-2 family members, including BCL-2 and BCL-XL, and evaluation of the combination in models that better represent tumor-specific biology and therapeutic response. Further work should also define how Golgi architectural perturbation engages apoptotic signaling and whether Aurora-A mislocalization contributes to that response. These studies will be required to assess the translational potential of Golgi checkpoint-targeted combinations.

## Figures and Tables

**Figure 1:**
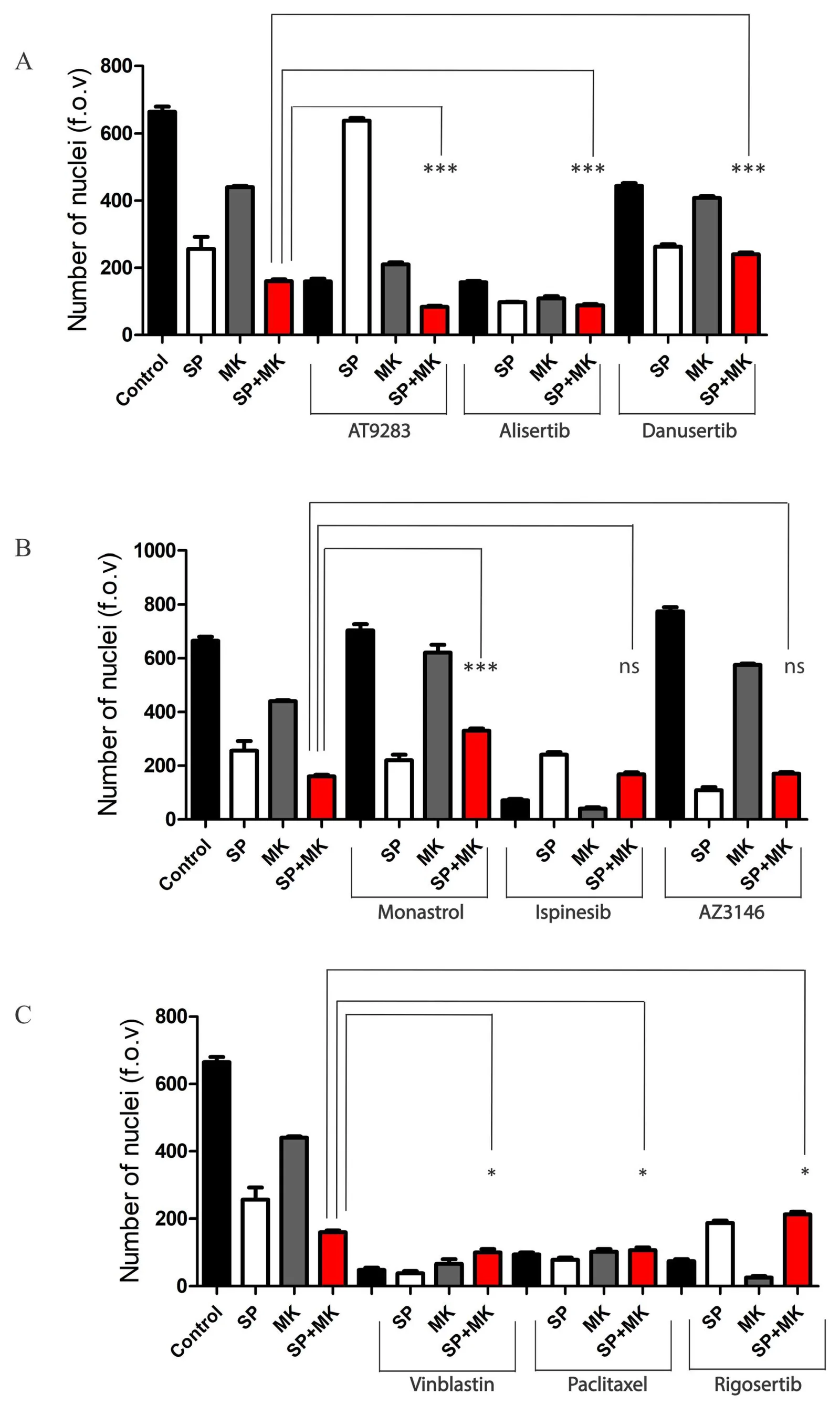
A compound screen identifies Navitoclax as a potent sensitizer of Golgi checkpoint-induced mitotic stress—nuclei count assay. HeLa cells were grown on fibronectin-coated coverslips, synchronized by double thymidine block, and treated overnight with SP600125 (25 µM) and MK1775 (0.3 µM) alone or in combination with each compound at the concentrations indicated in Table 1. Cells were fixed, stained with Hoechst 33342, and the number of nuclei per field of view (f.o.v.) was quantified by fluorescence microscopy. Data are expressed as mean ± SD from three independent experiments. Statistical analysis was performed using one-way ANOVA followed by Tukey’s multiple comparisons test; *p* values are indicated as follows: ns, not significant (*p* > 0.05); *, *p* < 0.05; ***, *p* ≤ 0.001. (**A**) SP+MK vs. AT9283+SP+MK ***, *p* ≤ 0.001; SP+MK vs. Alisertib+SP+MK ***, *p* ≤ 0.001; SP+MK vs. Danusertib+SP+MK ***, *p* ≤ 0.001. (**B**) SP+MK vs Monastrol+SP+MK ***, *p* ≤ 0.001; SP+MK vs. Ispinesib+SP+MK ns; SP+MK vs. AZ3146+SP+MK ns. (**C**) SP+MK vs. Vinblastine+SP+MK *, *p* < 0.05; SP+MK vs. Paclitaxel+SP+MK *, *p* < 0.05; SP+MK vs. Rigosertib+SP+MK *, *p* < 0.05.

**Figure 2:**
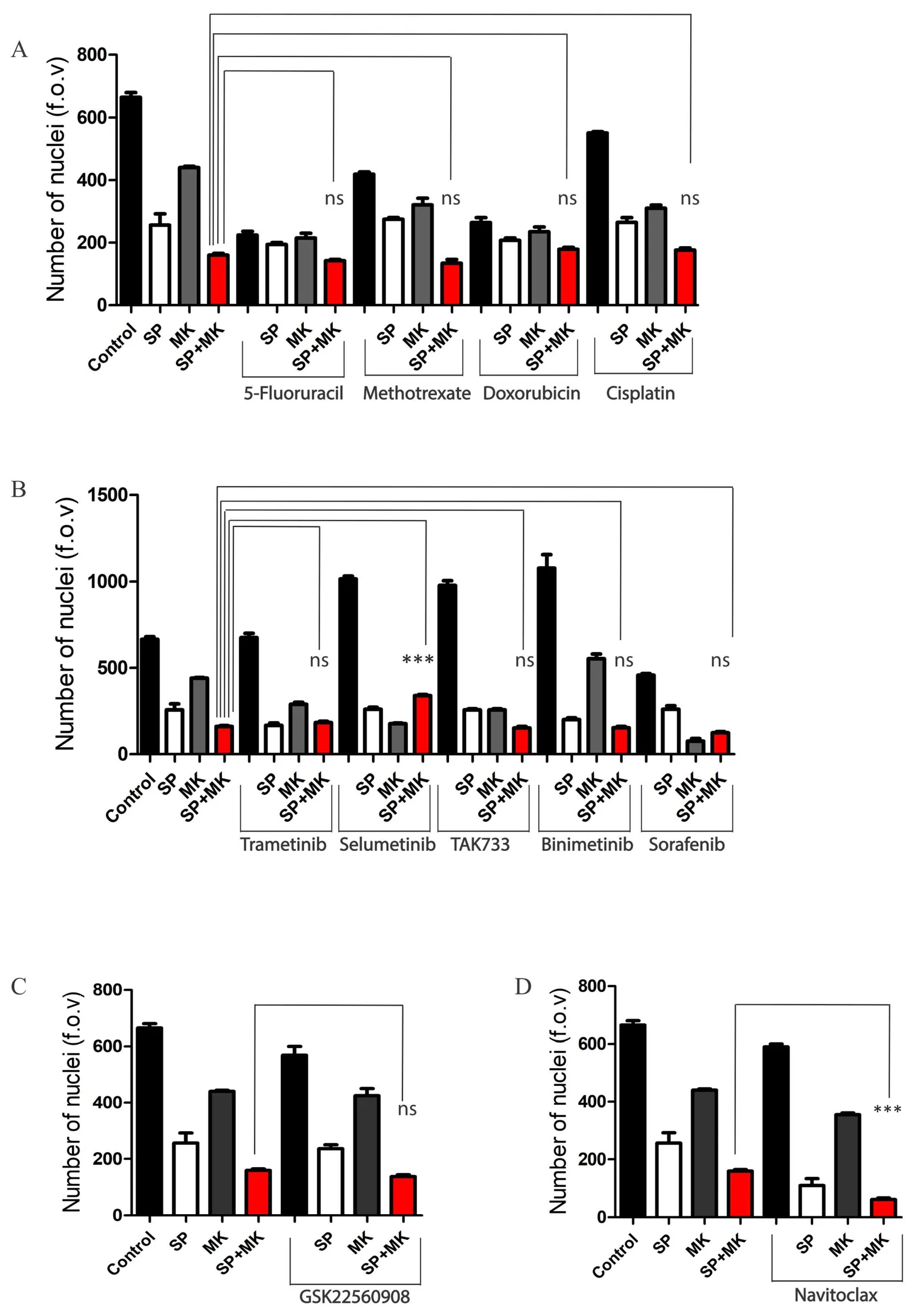
A compound screen identifies Navitoclax as a potent sensitizer of Golgi checkpoint-induced mitotic stress—nuclei count assay. Hela cells were treated as in Figure 1 alone or in combination with the other compounds listed in Table 1 at the indicated concentrations. Cells were fixed, stained with Hoechst 33342, and the number of nuclei per field of view (f.o.v.) was quantified by fluorescence microscopy. Data are expressed as mean ± SD from three independent experiments. Statistical analysis was performed using one-way ANOVA followed by Tukey’s multiple comparisons test; *p* values are indicated as follows: ns, not significant (*p* > 0.05); ***, *p* ≤ 0.001. (**A**) SP+MK vs. 5-Fluorouracil+SP+MK ns; SP+MK vs. Methotrexate+SP+MK ns; SP+MK vs. Doxorubicin+SP+MK ns; SP+MK vs. Cisplatin+SP+MK ns. (**B**) SP+MK vs. Trametinib+SP+MK ns; SP+MK vs. Selumetinib+SP+MK ***, *p* ≤ 0.001; SP+MK vs. TAK733+SP+MK ns; SP+MK vs. Binimetinib+SP+MK ns; SP+MK vs. Sorafenib+SP+MK ns. (**C**) SP+MK vs. GSK22560908+SP+MK ns. (**D**) SP+MK vs. Navitoclax+SP+MK ***, *p* ≤ 0.001.

**Figure 3:**
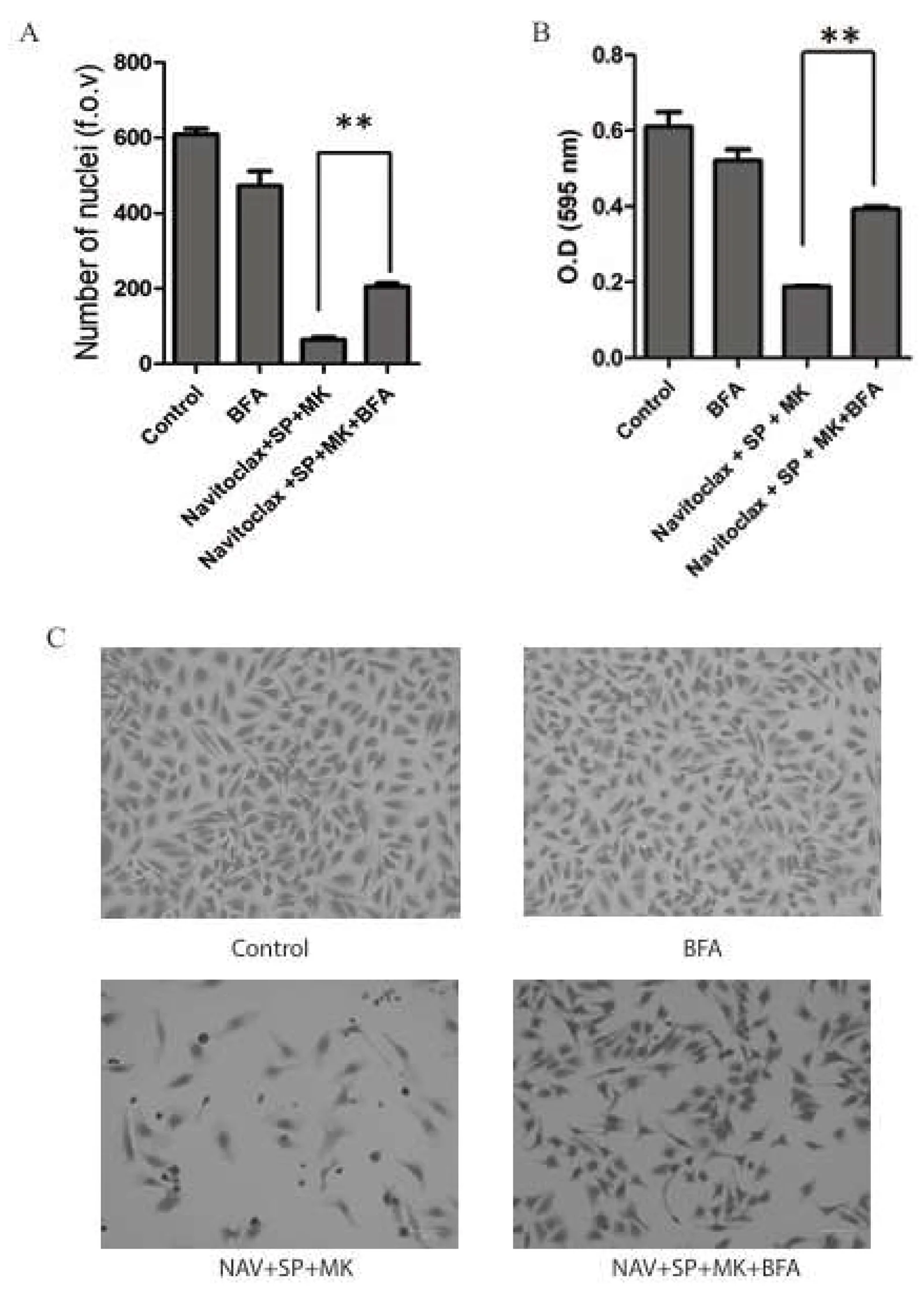
Brefeldin A-induced Golgi dispersal partially rescues cell viability in Navitoclax+SP+MK-treated cells. (**A**) Number of nuclei per field of view (f.o.v.) of HeLa cells treated with SP600125 (25 µM), MK1775 (0.3 µM), and Navitoclax (0.5 µM) in the presence or absence of Brefeldin A (200 ng/mL). Data are expressed as mean ± SD from three independent experiments. Statistical significance: Navitoclax+SP+MK vs. Navitoclax+SP+MK+BFA, ** *p* < 0.01 (one-way ANOVA with Sidak’s multiple comparisons test). (**B**) HeLa cells were seeded in 24-well plates, synchronized by double thymidine block, and treated overnight with SP600125 (25 µM) and MK1775 (0.3 µM) alone or in combination with each compound at the concentrations indicated in Table 1. Cell viability was assessed by crystal violet staining and absorbance measured at 595 nm. Data are expressed as mean ± SD from three independent experiments. Statistical significance: Navitoclax+SP+MK vs. Navitoclax+SP+MK+BFA, ** *p* < 0.01 (one-way ANOVA with Sidak’s multiple comparisons test). (**C**) Representative image of crystal violet staining from the experiment shown in (**B**).

**Figure 4:**
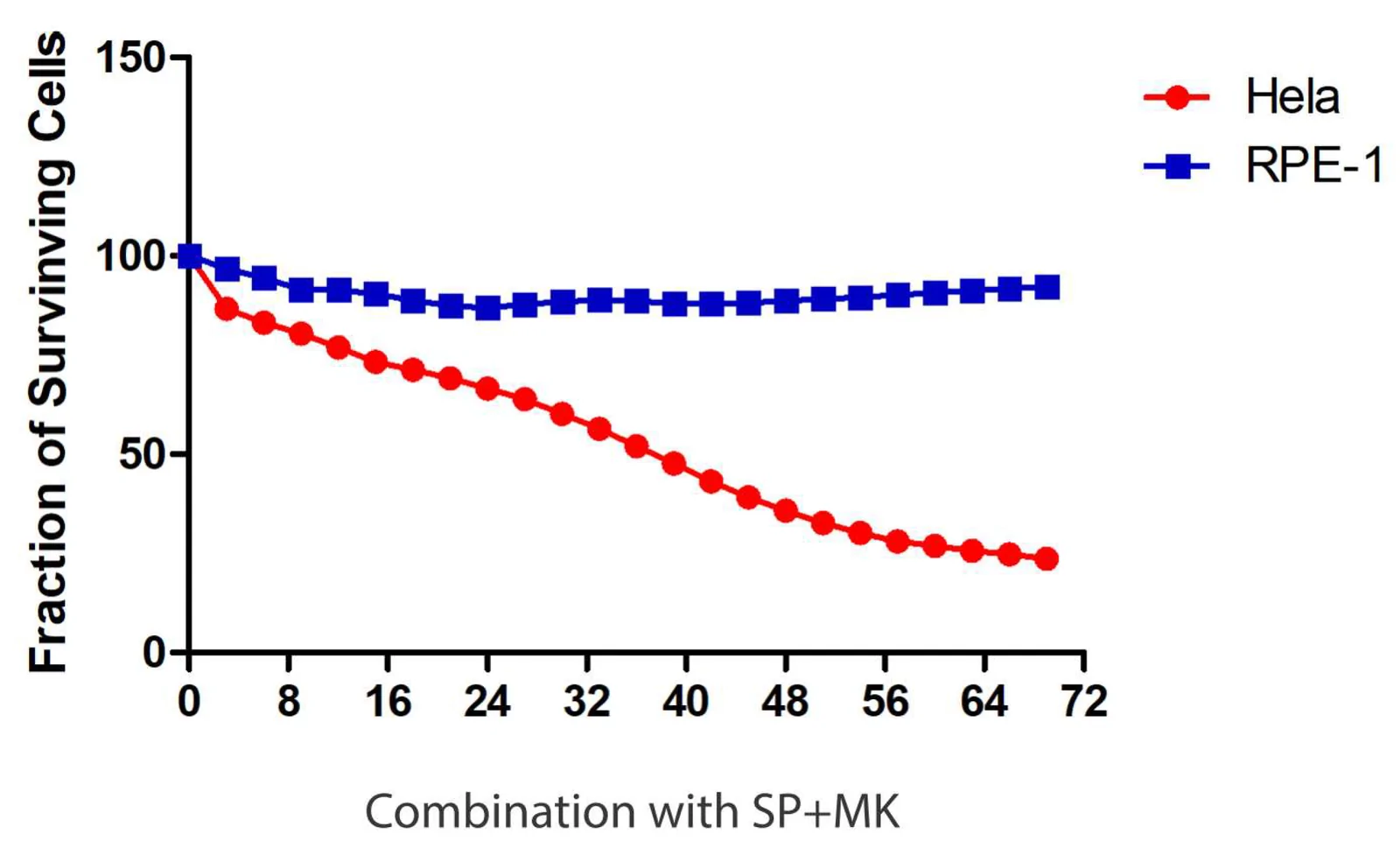
Tumor cells are selectively sensitive to SP+MK-induced mitotic stress. Time-course survival analysis of HeLa and RPE-1 cells treated with SP600125 (25 µM) and MK1775 (0.3 µM) using the IncuCyte S3 Live-Cell Analysis System over a 72 h period. Data are expressed as mean ± SD from three independent experiments. Cell confluence was quantified using the integrated IncuCyte S3 software and normalized to the initial value at time zero to account for plating variations.

**Figure 5:**
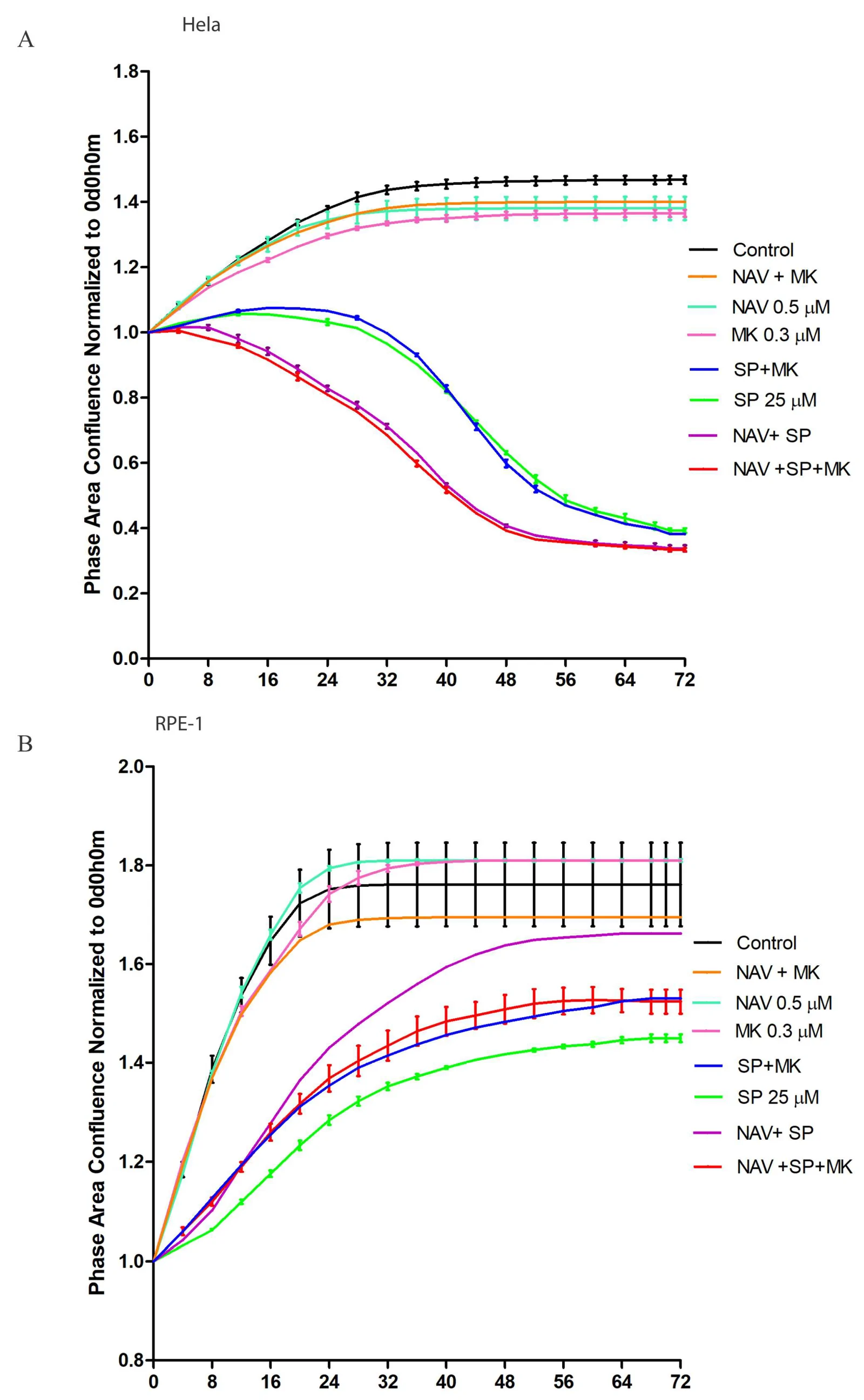
Navitoclax selectively potentiates SP+MK-induced antiproliferative activity in HeLa but not RPE-1 cells. Proliferation of HeLa (**A**) and RPE-1 (**B**) cells treated with SP600125 (25 µM), MK1775 (0.3 µM), and Navitoclax (0.5 µM), alone or in combination, monitored using the IncuCyte S3 Live-Cell Analysis System over a 72 h period. Data are normalized to the initial value at time zero to account for plating variations. SP+MK vs. Navitoclax+SP+MK in HeLa cells.

**Figure 6:**
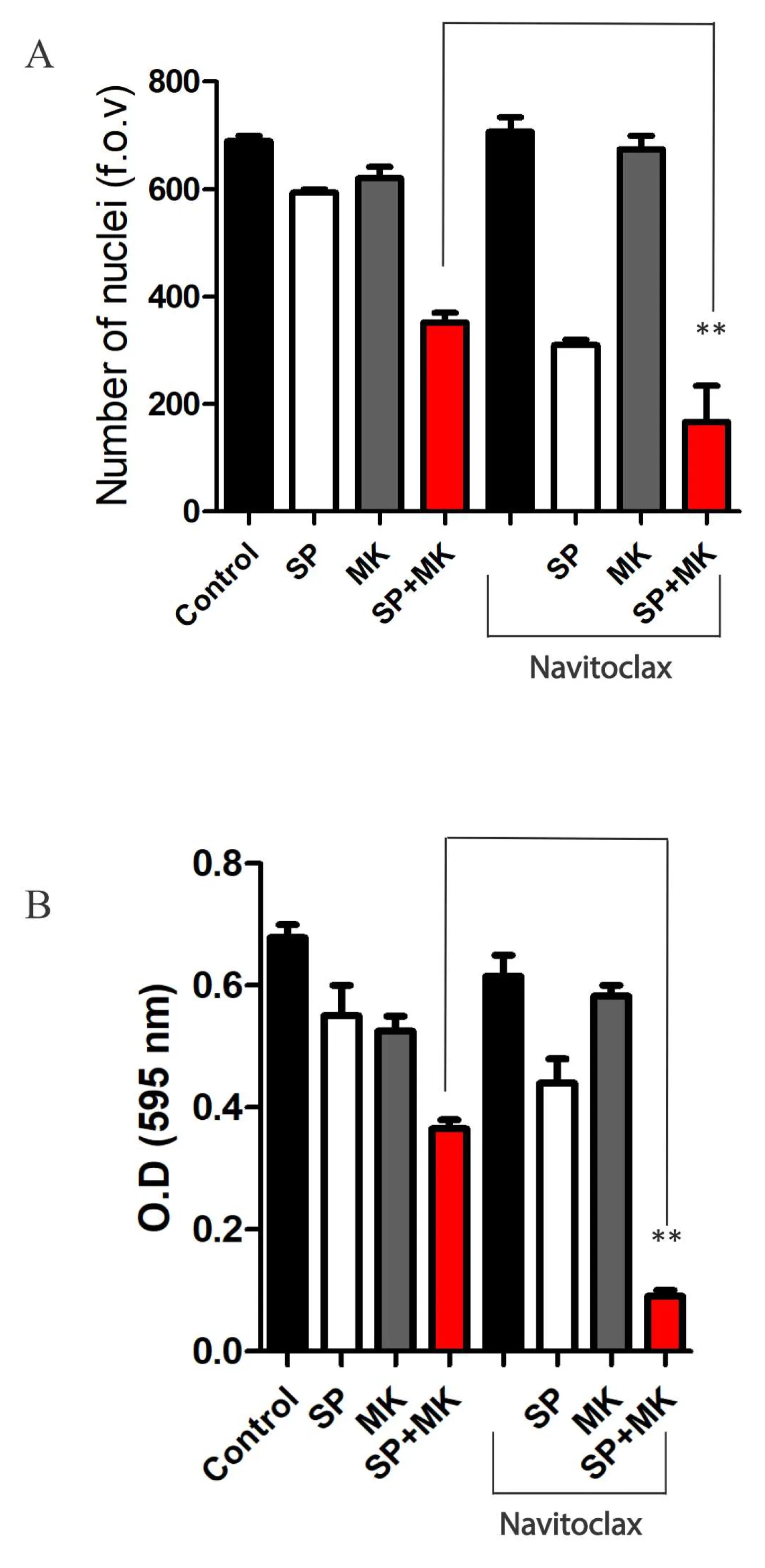
Reduced concentrations of SP600125 and MK1775 combined with Navitoclax retain antiproliferative efficacy in HeLa cells. (**A**) HeLa cells were grown on fibronectin-coated coverslips, synchronized by double thymidine block, and treated overnight with SP600125 (10 µM) and MK1775 (0.1 µM) alone or in combination with Navitoclax (0.5 µM). Cells were fixed, stained with Hoechst 33342, and the number of nuclei per field of view (f.o.v.) was quantified. Data are expressed as mean ± SD from three independent experiments. SP+MK vs. Navitoclax+SP+MK: ** *p* < 0.01. (**B**) Crystal violet staining of HeLa cells treated as described in A. Absorbance measured at 595 nm. Data are expressed as mean ± SD from three independent experiments. SP+MK vs. Navitoclax+SP+MK: ** *p* < 0.01.

**Figure 7:**
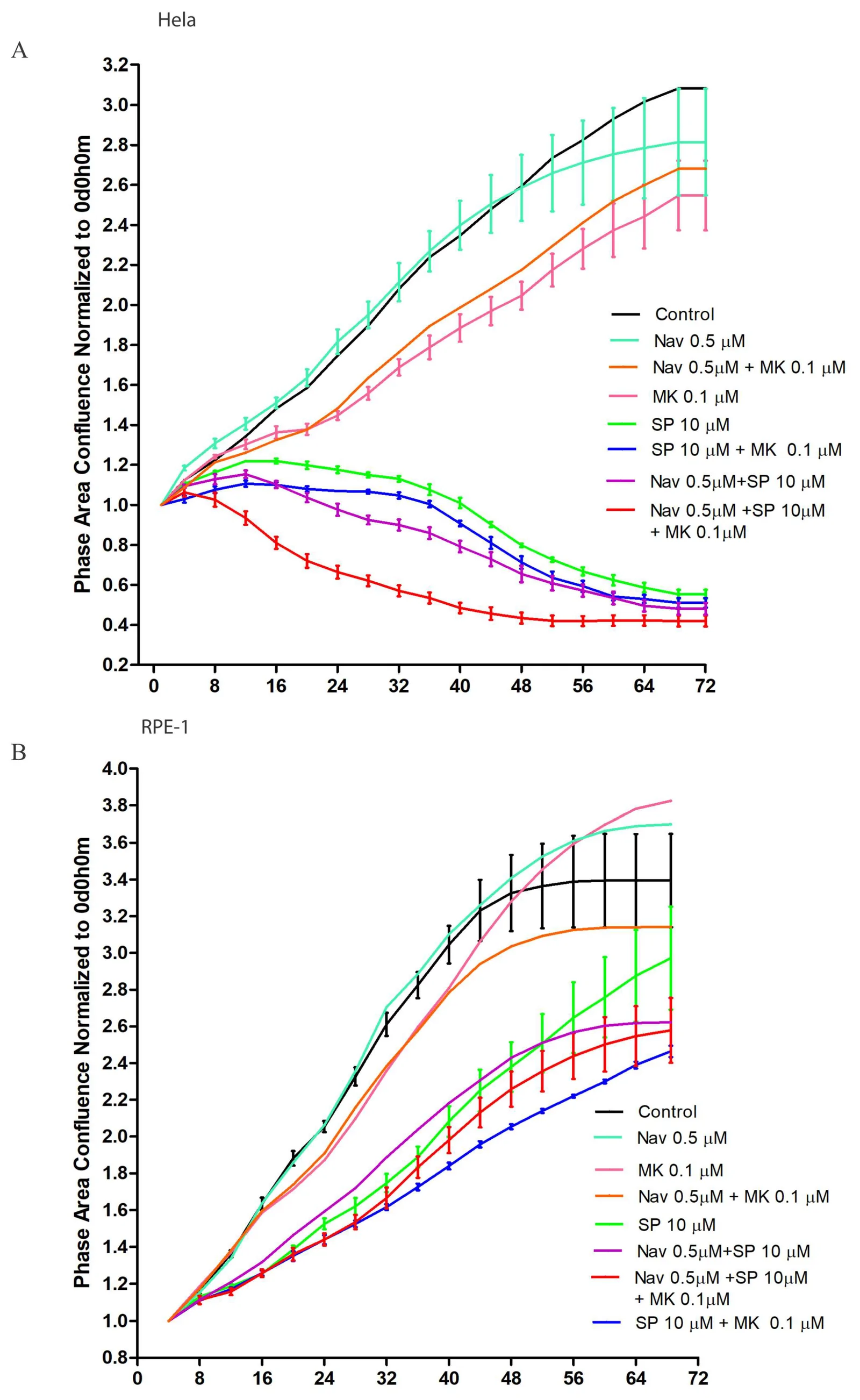
Reduced concentrations of SP600125 and MK1775 combined with Navitoclax selectively reduce proliferation in HeLa but not RPE-1 cells. Proliferation of HeLa (**A**) and RPE-1 (**B**) cells treated with SP600125 (10 µM), MK1775 (0.1 µM), and Navitoclax (0.5 µM), alone or in combination, monitored using the IncuCyte S3 Live-Cell Analysis System over a 72 h period. Data are expressed as mean ± SD from three independent experiments. Cell confluence was quantified using the integrated IncuCyte S3 software and normalized to the initial value at time zero to account for plating variations. SP+MK vs. Navitoclax+SP+MK in HeLa cells.

**Figure 8:**
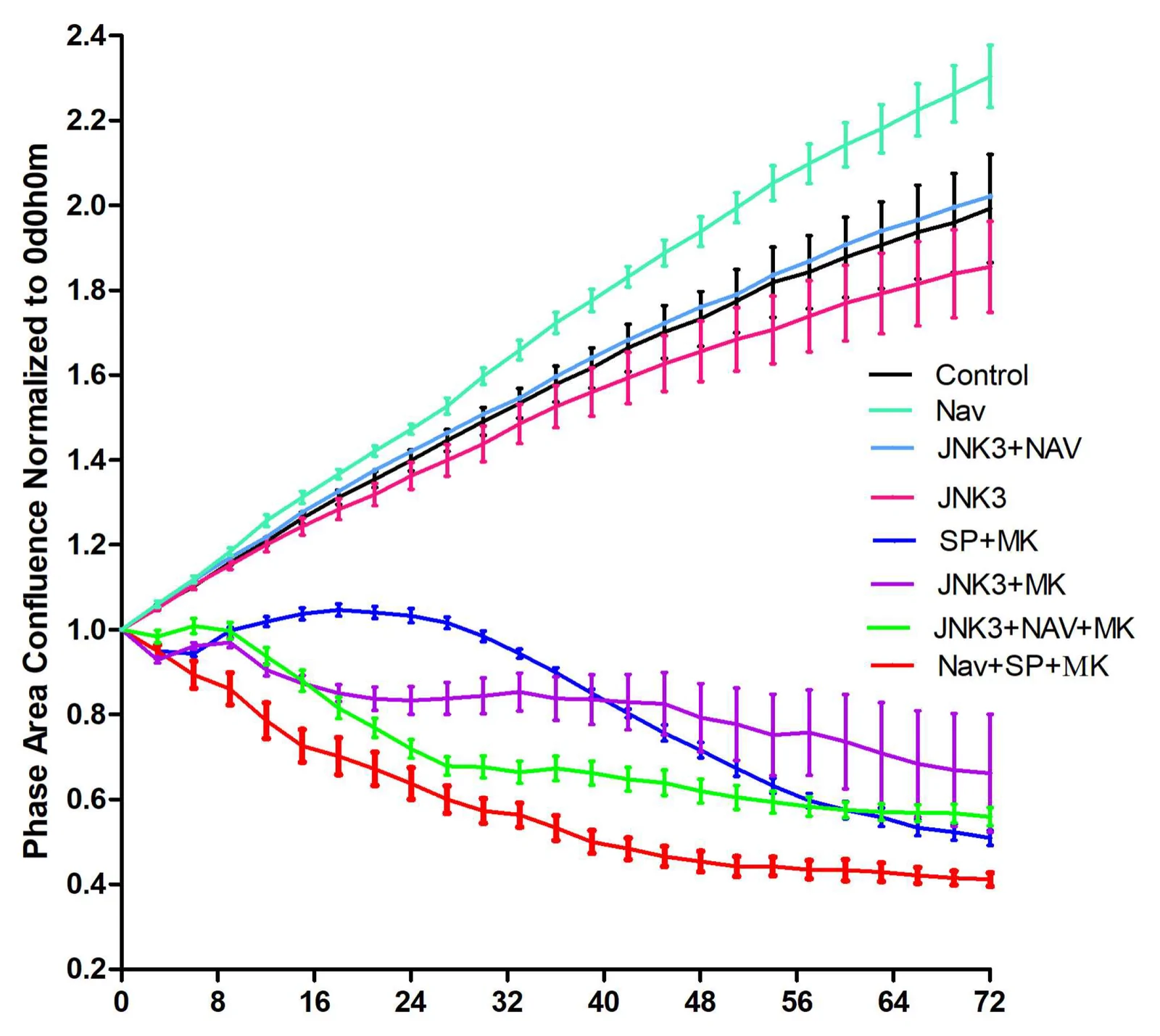
The antiproliferative response to the triple combination is linked to the degree of Golgi compaction. Proliferation of HeLa cells treated with either SP600125 (25 µM) or JNK inhibitor III (5 µM), in combination with MK1775 (0.3 µM) and Navitoclax (0.5 µM), monitored using the IncuCyte S3 Live-Cell Analysis System over a 72 h period. Data are expressed as mean ± SD from three independent experiments. Cell confluence was quantified using the integrated IncuCyte S3 software and normalized to the initial value at time zero to account for plating variations. Navitoclax+SP+MK vs. Navitoclax+JNK inhibitor III+MK.

**Figure 9:**
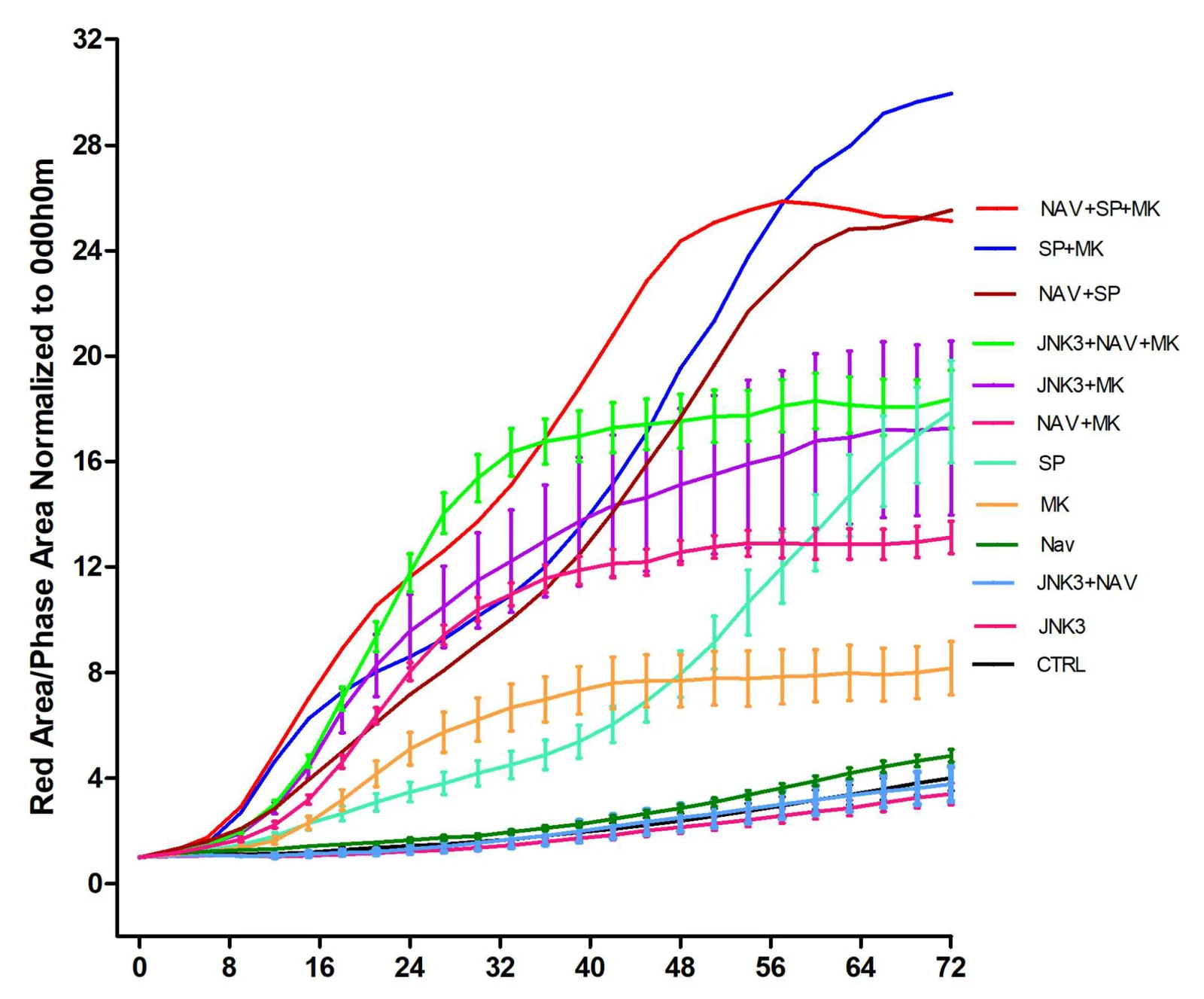
Navitoclax shifts the cellular response to SP+MK from mitotic slippage toward apoptotic cell death. Time-course analysis of apoptotic activity in HeLa cells treated with Navitoclax (0.5 µM), SP600125 (25 µM), MK1775 (0.3 µM), and JNK inhibitor III (5 µM), alone or in combination. Four hours after drug treatment, Annexin V NIR Dye (1 μg/mL) was added to the culture medium. Phase-contrast and NIR fluorescence images were acquired every 4 h for 72 h using the IncuCyte S3 Live-Cell Analysis System. Apoptotic activity is expressed as the ratio of Annexin V NIR-positive area to phase-contrast area, normalized to time zero (mean ± SEM, three independent experiments). Control vs. Navitoclax+SP+MK.

## Data Availability

The original contributions presented in this study are included in the article. Further inquiries can be directed to the corresponding authors.

## Supplementary Materials

The following supporting information can be downloaded at: https://www.mdpi.com/article/10.3390/cells15191825/s1, Figure S1: Navitoclax selectively enhances the antiproliferative activity of SP+MK in HeLa but not RPE-1 cells; Figure S2: Navitoclax+SP+MK selectively reduces survival in HeLa but not RPE-1 cells; Figure S3: The antiproliferative activity of the Navitoclax+SP+MK combination is maintained at lower drug concentrations; Figure S4: The efficacy of the triple combination Navitoclax+SP+MK is linked to the degree of Golgi compaction; Figure S5: Navitoclax promotes apoptotic cell death in SP+MK-sensitized cells.

## Abbreviations

The following abbreviations are used in this manuscript:

ANOVA	Analysis of Variance
BCL-2	B-cell lymphoma 2
BCL-xL	B-cell lymphoma-extra large
BFA	Brefeldin A
CDK1	Cyclin-Dependent Kinase 1
DHFR	Dihydrofolate Reductase
DMEM	Dulbecco’s Modified Eagle Medium
DMSO	Dimethyl Sulfoxide
DNA	Deoxyribonucleic Acid
ERK	Extracellular Signal-Regulated Kinase
FAK	Focal Adhesion Kinase
FBS	Fetal Bovine Serum
f.o.v.	Field of View
Golgi	Golgi Apparatus
GRASP65	Golgi Reassembly and Stacking Protein 65
JNK	c-Jun N-terminal Kinase
KSP	Kinesin Spindle Protein (Eg5/KIF11)
MAPK	Mitogen-Activated Protein Kinase
MEK	Mitogen-Activated Protein Kinase Kinase
MK	MK1775 (Wee1 inhibitor)
MT	Microtubules
PBS	Phosphate-Buffered Saline
PLK1	Polo-Like Kinase 1
RPE-1	hTERT-immortalized Retinal Pigment Epithelial cells
SAC	Spindle Assembly Checkpoint
SD	Standard Deviation
SEM	Standard Error of the Mean
SP	SP600125 (JNK inhibitor)
TS	Thymidylate Synthase
Wee1	Wee1 Kinase
