# Supplementary material for: Pharmacological Perturbation of the Golgi Checkpoint Synergizes with Apoptotic Priming to Selectively Induce Tumor Cell Death
Source: Cells. 2026 Oct 7;15(19):1825. doi: 10.3390/cells15191825 (PMC13664185; doi:10.3390/cells15191825)
Supplement: Supplementary file 1 [file cells-15-01825-s001.zip › cells-4568570-supplementary.pdf]

A. Hela

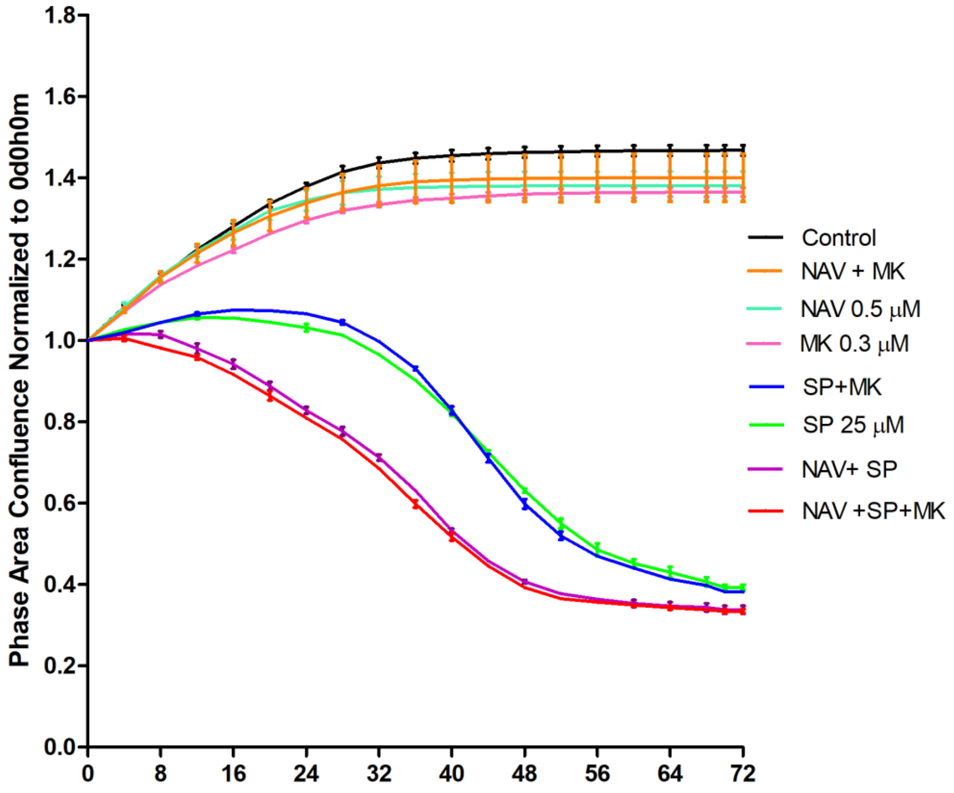

B. RPE-1

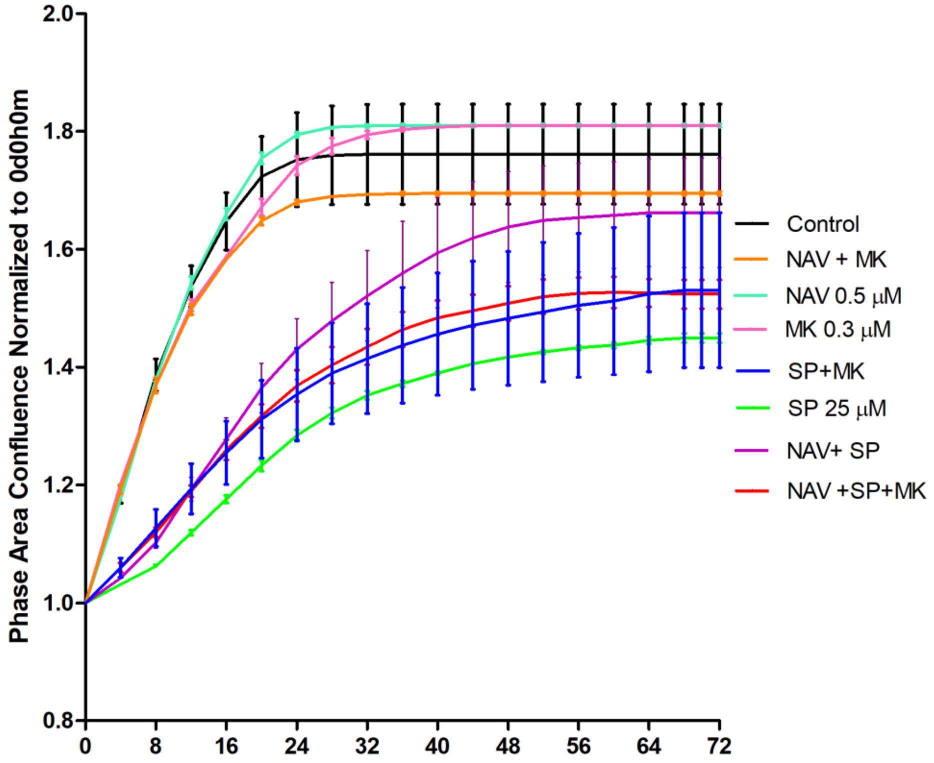

Figure S1. Navitoclax selectively enhances the antiproliferative activity of SP+MK in HeLa but not RPE-1 cells. The graphs shown here include all standard deviation (SD) error bars for the data presented in Figure 5, including those omitted in the main figure for improved readability.

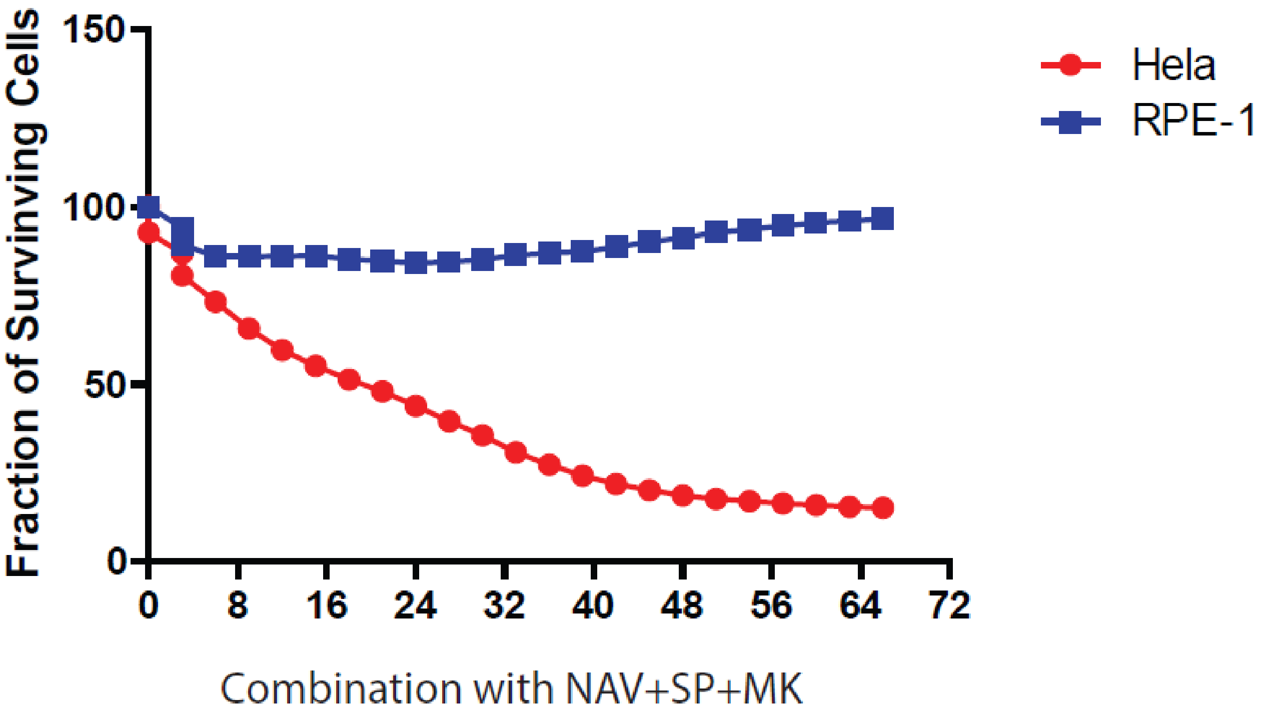

Figure S2. Navitoclax+SP+MK selectively reduces survival in HeLa but not RPE-1 cells. Time-course survival analysis of HeLa and RPE-1 cells treated with SP600125 (25 μM), MK1775 (0.3 μM), and Navitoclax (0.5 μM) using the IncuCyte S3 Live-Cell Analysis System over a 72-hour period. Data are expressed as mean ± SD from three independent experiments. Cell confluence was quantified using the integrated IncuCyte S3 software and normalized to the initial value at time zero to account for plating variations.

A. Hela

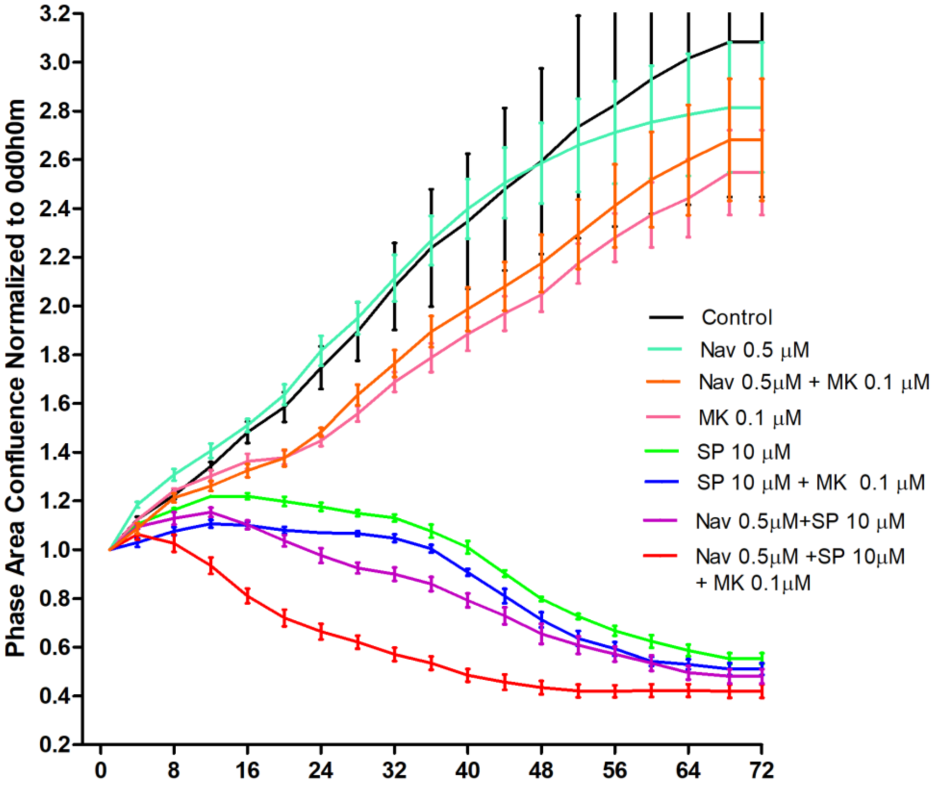

B. RPE-1

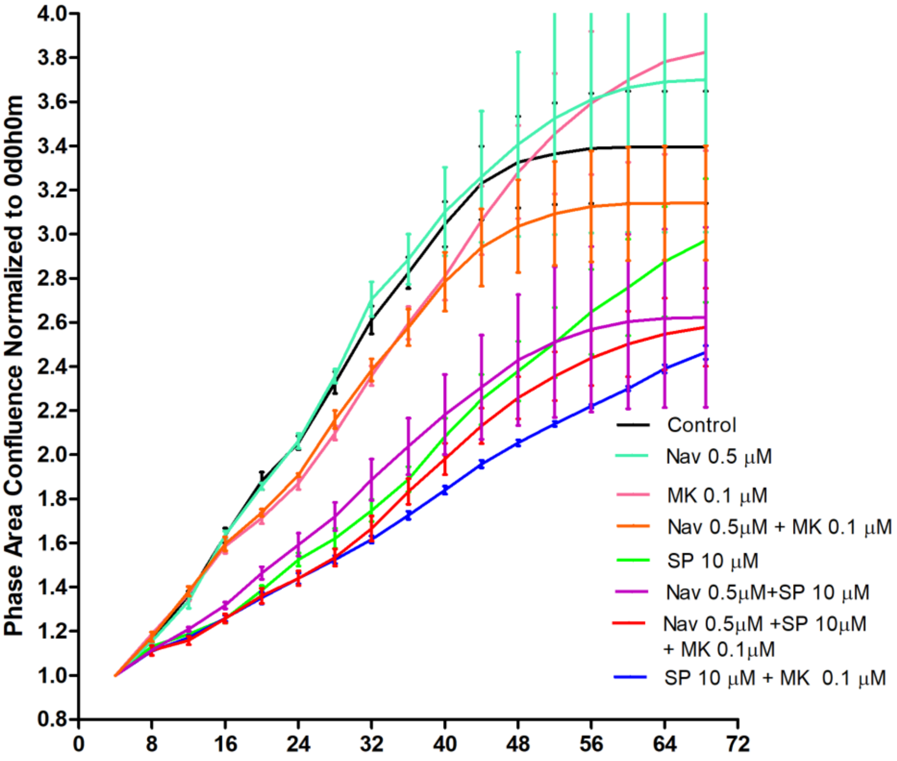

Figure S3. The antiproliferative activity of the Navitoclax+SP+MK combination is maintained at lower drug concen-trations. The graphs shown here include all standard deviation (SD) error bars for the data presented in Figure 7, including those omitted in the main figure for improved readability.

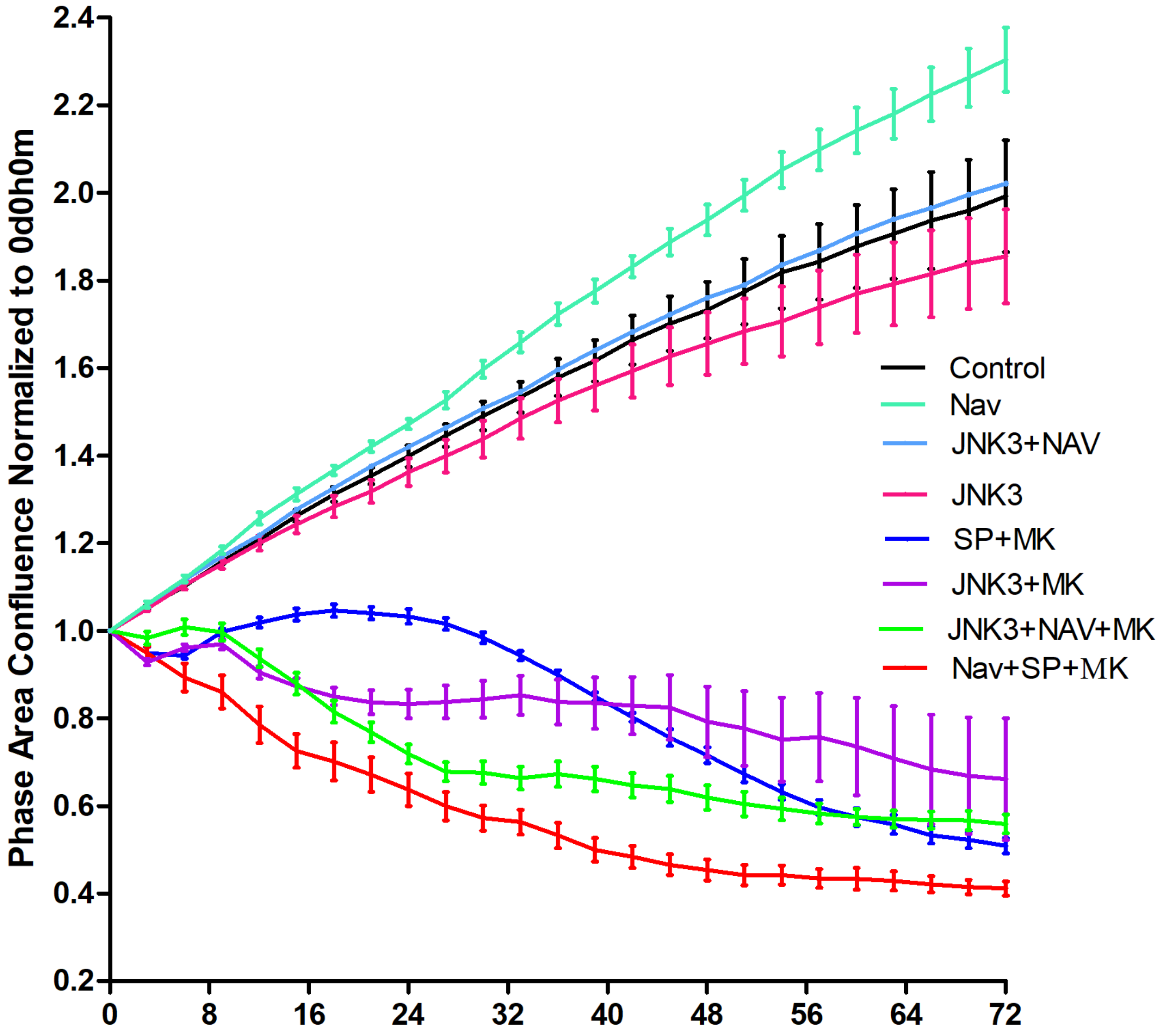

Figure S4. The efficacy of the triple combination Navitoclax+SP+MK is linked to the degree of Golgi compaction. The graphs shown here include all standard deviation (SD) error bars for the data presented in Figure 8, including those omitted in the main figure for improved readability.

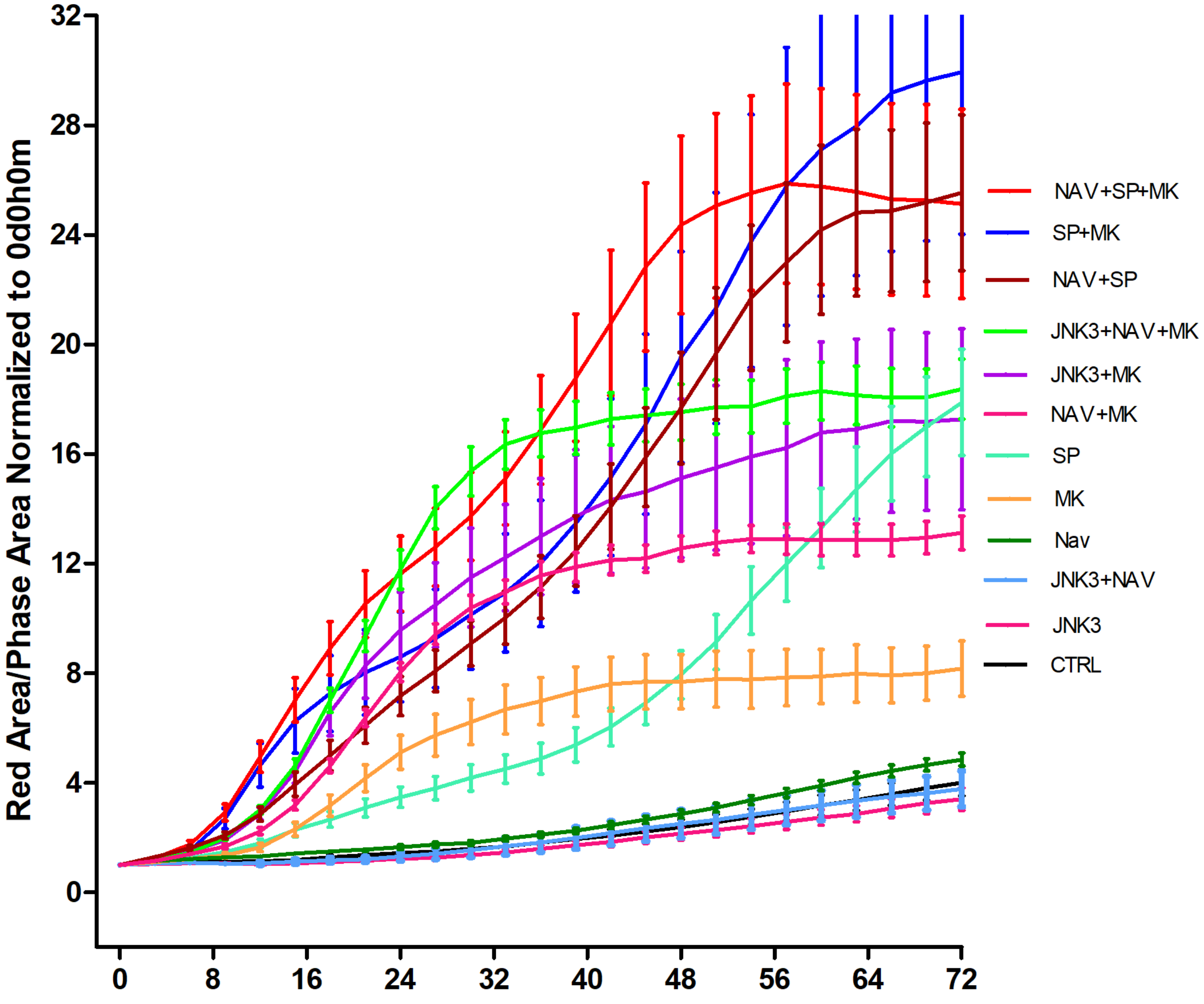

Figure S5. Navitoclax promotes apoptotic cell death in SP+MK-sensitized cells. The graphs shown here include all standard deviation (SD) error bars for the data presented in Figure 9, including those omitted in the main figure for improved read-ability.
